# Consolidation of auditory fear memories formed by weak unconditioned stimuli requires NMDA receptor activation and *de novo *protein synthesis in the striatum

**DOI:** 10.1186/1756-6606-6-17

**Published:** 2013-04-15

**Authors:** Ayumi Kishioka, Takeshi Uemura, Fumiaki Fukushima, Masayoshi Mishina

**Affiliations:** 1 Department of Molecular Neurobiology and Pharmacology, Graduate School of Medicine, University of Tokyo, Tokyo 113-0033, Japan; 2 Department of Molecular and Cellular Physiology, School of Medicine, Shinshu University, Nagano, 390-8621, Japan; 3 Brain Science Laboratory, The Research Organization of Science and Technology, Ritsumeikan University, Shiga 525-8577, Japan

**Keywords:** Memory, Striatum, Auditory fear conditioning, NMDA receptor, Protein synthesis

## Abstract

**Background:**

Fear is one of the most potent emotional experiences and is an adaptive component of response to potentially threatening stimuli. Cumulative evidence suggests that the amygdala plays a central role in the acquisition, storage and expression of fear memory. We previously showed that the selective ablation of striatal neurons in the adult brain impairs the long-term, but not short-term, memory for auditory fear conditioning with a lower-intensity footshock. This finding raises an intriguing possibility that long-term auditory fear memory may be consolidated in the striatum.

**Results:**

There was a significant difference in the freezing responses between two groups of mice subjected to paired and unpaired conditioning, indicating that the auditory fear conditioning with a lower-intensity footshock is an associative learning. Post-conditioning infusion of NMDA receptor inhibitors into the striatum suppressed the consolidation of auditory fear memory when mice were conditioned with a low-intensity footshock. Furthermore, intra-striatum infusion of protein synthesis blocker anisomycin immediately or 1 h after the conditioning prevented the formation of auditory fear memory. On the other hand, the infusion of anisomycin 3 h after conditioning exerted little effect on the auditory fear conditioning, consistent with the presence of a critical time window of protein synthesis for memory consolidation.

**Conclusions:**

These results suggest that NMDA receptors and *de novo* protein synthesis in the striatum are crucial for the consolidation of auditory fear memory formed with a low-intensity unconditioned stimulus.

## Background

Fear is one of the most potent emotional experiences of our lifetime and is an adaptive component of response to potentially threatening stimuli, serving a function that is critical to the survival of higher vertebrates [1,2]. Animals can learn that specific sensory cues in the environment predict aversive events through a form of associative learning termed fear conditioning. The memory of learned fear can be assessed quantitatively using a Pavlovian fear-conditioning paradigm [3]. The amygdala plays an essential role in the acquisition, storage and expression of fear memories [1-4]. Blockade of *N*-methyl-D-aspartate (NMDA) receptors in the amygdala suppresses the acquisition of auditory fear conditioning [5,6], while the formation of long-term auditory fear memory can be disrupted by the treatment of protein synthesis inhibitors [7].

The striatum is regarded as a key forebrain structure underlying appetitive learning and memory [8]. NMDA receptors in the striatum are required for the consolidation of appetitive Pavlovian learning [9,10] and the acquisition of instrumental learning [11,12]. *De novo* protein synthesis in the striatum is also required for the consolidation of appetitive Pavlovian and instrumental learning [13,14]. Previously, we developed an inducible striatal neuron ablation system using transgenic mice and revealed that the ablation of striatal neurons induced in the adult brain impaired the formation of long-term, but not short-term, auditory fear memory when conditioned with a weak unconditioned stimulus (US) [15]. Furthermore, post-conditioning ablation of striatal neurons after memory formation diminished the auditory fear memory [15]. These findings raise an intriguing possibility that long-term auditory fear memory may be consolidated at least partly in the striatum. Here, we examined the issue by administration of NMDA receptor antagonists and protein synthesis inhibitor into the striatum during auditory fear conditioning. Our results showed that the consolidation of auditory fear memories formed with a low-intensity US required post-conditioning NMDA receptor activation and *de novo* protein synthesis in the striatum.

## Results

### Auditory fear conditioning with a low-intensity footshock

**Figure 1:**
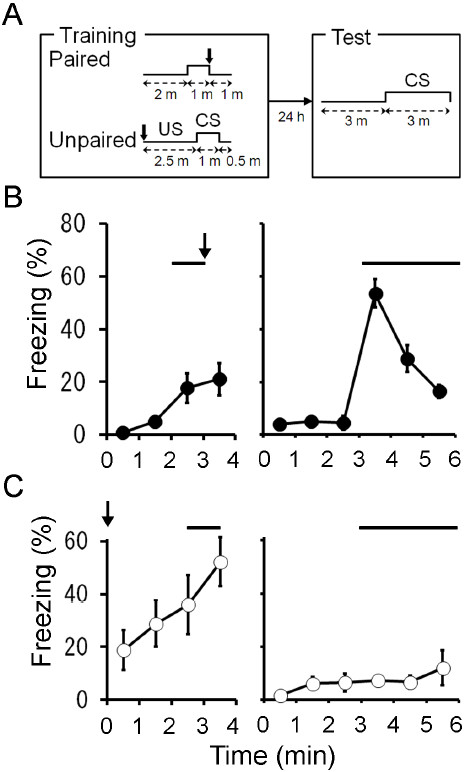
**Auditory fear conditioning with a lower-intensity footshock is associative in nature. **(**A**) Schema of fear conditioning in the paired or unpaired paradigm. In the paired conditioning paradigm, mice were placed in the conditioning chamber for 2 min and then presented with a tone for 1 min. At the end of the tone presentation, the mice were given a scrambled electrical footshock (0.3 mA, 1 s). One minute after footshock, the mice were returned to their home cages. In the unpaired conditioning paradigm, mice received the footshock immediately after placement in the conditioning chamber. Two and half minutes after the placement, a tone was presented for 1 min. Half minute later, mice were returned to their home cages. Twenty-four hours after conditioning, mice were placed in a novel chamber for 3 min and then the tone was presented for 3 min. (**B**) Freezing responses of paired group (filled circles, *n *= 7) on the conditioning (left) and test (right) days. (**C**) Freezing responses of unpaired group (open circles, *n *= 6) on the conditioning (left) and test (right) days. Arrows and solid lines indicate footshock and tone, respectively.

We compared the freezing responses of mice in paired and unpaired paradigms with a low-intensity US (Figure 1A). In the paired paradigm, mice were placed in a conditioning chamber for 2 min and then a tone (65 dB, 10 kHz) was presented for 1 min. At the end of the tone presentation, the mice were given a low-intensity footshock (0.3 mA, 1 s). In the unpaired paradigm, the low-intensity footshock was given immediately after mice were placed in the conditioning chamber and 2.5 min after the placement, the tone was presented for 1 min. Twenty-four hours later, the animals were placed in a novel chamber for 3 min and then the tone was presented for 3 min. Mice conditioned with the paired paradigm showed strong freezing responses upon tone presentation (Figure 1B). In contrast, mice given unpaired conditioning exhibited little freezing response to the tone (Figure 1C). There was a significant difference in the freezing responses between two groups of mice (*F*_1,11_ = 40.3, *P* < 0.001, *n* = 6 (paired) or 7 (unpaired), repeated measures ANOVA). We thus confirmed that the auditory fear conditioning with a lower-intensity footshock is an associative learning.

### Intra-striatum infusion of APV impaired the formation of auditory fear memory

**Figure 2:**
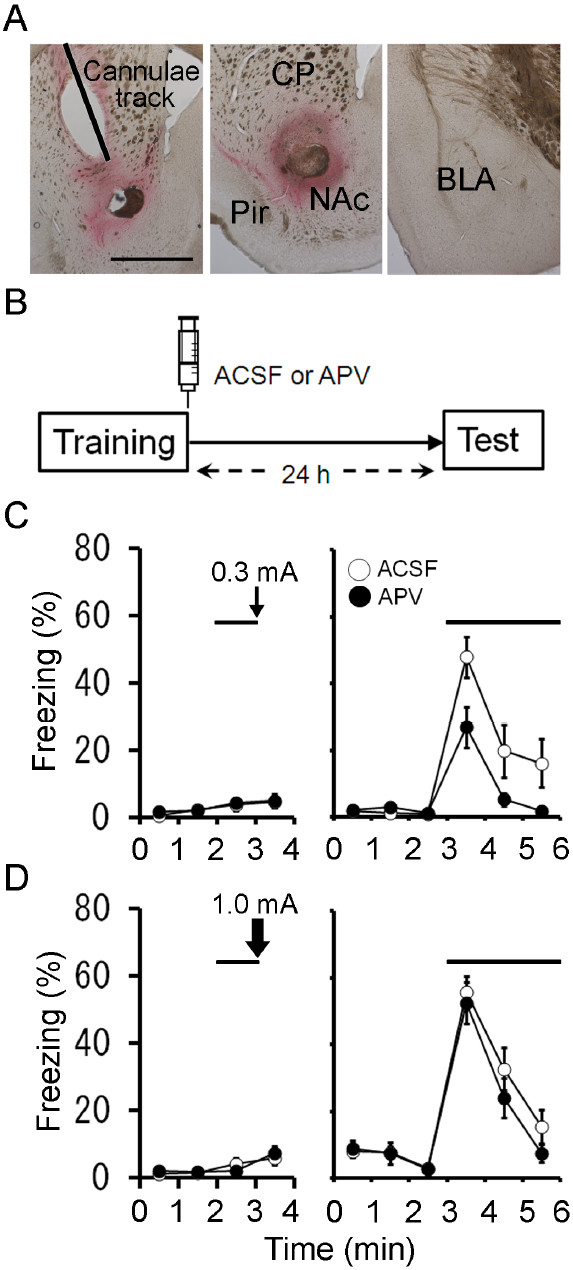
**Post-training infusion of APV into the striatum impaired long-term fear memory.** (**A**) Examination of dye spread in the striatum. Bright field photomicrograph of colonal brain sections showing the spread of the dye solution (0.5 μl) in the striatum. An angled line indicates a track of guide cannula. (**Left**) Dye solution spreads largely in the NAc and along the cannula track in the overlying CP. (**Middle**) Dye solution in the NAc usually spreads vertically as well (*n* = 6). (**Right**) Dye solution did not reach the BLA or CeA. Scale bar: 1 mm. (**B**) Experimental design. Mice were injected with 0.5 μl of ACSF or APV into the striatum immediately after auditory fear conditioning with a lower-intensity footshock, and tested 24 h later. (**C**) Freezing responses of ACSF (*n *= 10) and APV (*n *= 10) groups on the conditioning (left) and test (right) days. A solid line and a thin arrow represent tone and footshock (0.3 mA), respectively. (**D**) Freezing responses of ACSF (*n *= 15) and APV (*n *= 12) groups on the conditioning (left) and test (right) days. A solid line and a thick arrow represent tone and footshock (1.0 mA), respectively.

NMDA receptors are critically involved in learning and memory [16]. Here, we tested whether the activation of striatal NMDA receptors occurs during the consolidation period after the conditioning event. Mice received intra-striatum cannula implantations several days before behavioral experiments. We first monitored the dye spread in intra-striatum infusions. Dye solution (0.5 μl) spreads largely in the nucleus accumbens (NAc) and along the cannula track in the overlying caudate putamen (CP) (Figure 2A). Dye solution in the NAc usually spreads 1.07 ± 0.08 mm laterally and 1.31 ± 0.11 mm vertically (*n* = 6). It should be noted that the dye did not spread to the basolateral amygdala (BLA) and central amygdala (CeA), the crucial structures in auditory fear conditioning (Figure 2A).

Mice were trained using the auditory fear conditioning with a low-intensity footshock (0.3 mA) as above, and immediately after conditioning, they received intra-striatum infusion of NMDA receptor antagonist APV (1 μg in 0.5 μl) [1,17] (Figure 2B). When tested 24 h after conditioning, the freezing levels of APV-treated group were significantly smaller than those of ACSF-treated one (Figure 2C; *F*_1,18_ = 5.3, *P* = 0.034, *n* = 10 each, repeated measures ANOVA). Thus, the post-training infusion of APV in the stratum disrupted the formation of auditory fear memory.

Induced ablation of striatal neurons impaired the formation of long-term auditory fear memory when mice were conditioned with a lower-intensity, but not high-intensity, footshock [15]. Mice were trained with a high-intensity footshock (1.0 mA), and then received intra-striatum infusion of APV (Figure 2D). Twenty-four hours after the conditioning, the freezing levels were comparable between ACSF- and APV-treated groups (Figure 2D; *F*_1,25_ = 0.76, *P* = 0.39, ACSF, *n* = 15, APV, *n* = 12, repeated measures ANOVA). There were no significant differences in the freezing levels during tone presentation between ACSF-treated groups with low and high intensity footshocks (Figure 2C,D; *F*_1,23_ = 0.73, *P* = 0.40, ACSF-0.3 mA, *n* = 10 (0.3 mA) or 15 (1.0 mA), repeated measures ANOVA). Thus, striatal NMDA receptors are required for the formation of auditory fear memory with a low-intensity footshock but not for the formation of auditory fear memory with a high-intensity footshock.

**Figure 3:**
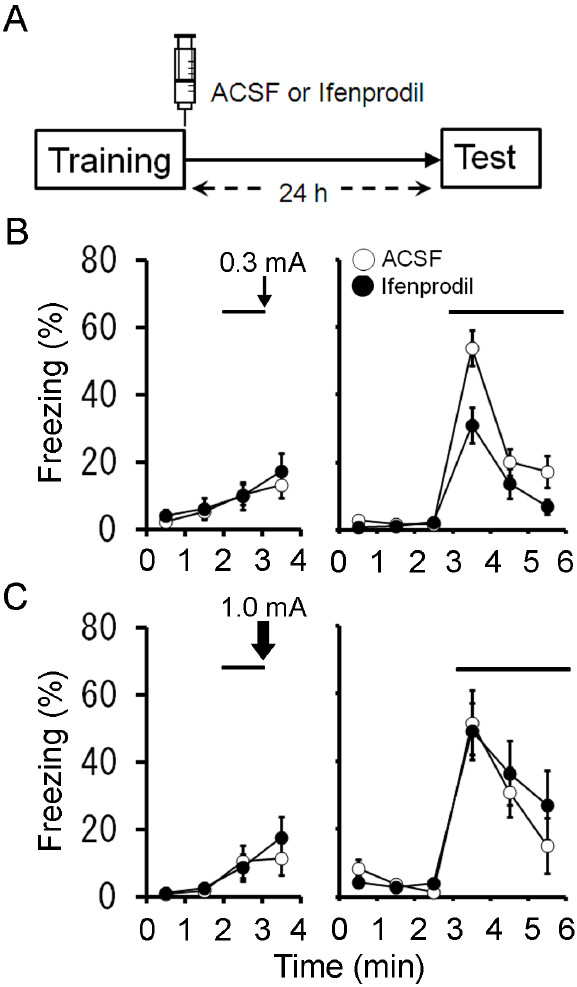
**Impairment of long-term auditory fear memory by post-training intra-striatum infusion of ifenprodil. **(**A**) Experimental design. Mice were injected with 0.5 μl of ACSF or ifenprodil into the striatum immediately after auditory fear conditioning with a footshock at 0.3 mA or 1.0 mA, and tested 24 h later. (**B**) Freezing responses of ACSF (*n *= 17) and ifenprodil (*n* = 16) groups conditioned with a low-intensity footshock (0.3 mA) on the conditioning (left) and test (right) days. There was a significant difference in the extent of the freezing responses between ACSF- and ifenprodil-treated mice (*F*_1,31 _= 7.19, *P *= 0.01, repeated measures ANOVA). (**C**) Freezing responses of ACSF (*n *= 9) and APV (*n *= 9) groups conditioned with a footshock at 1.0 mA on the conditioning (left) and test (right) days. The freezing responses of two groups of mice were comparable (*F*_1,16 _= 0.12, *P *= 0.74, repeated measures ANOVA). Solid lines and thick arrows represent tone and footshock, respectively.

We further tested the effect of GluRε2/GluN2B-selective NMDA receptor antagonist ifenprodil [18] on the formation of auditory fear memory. Immediately after auditory fear conditioning with a lower-intensity footshock (0.3 mA), mice received intra-striatum infusion of ACSF or ifenprodil (1 μg in 0.5 μl; see [19]). There was a significant difference in the extent of the freezing responses between ACSF- and ifenprodil-treated mice (Figure 3A, B). When conditioned with a high-intensity footshock (1.0 mA), however, the freezing responses of both groups of mice were comparable (Figure 3C). These results further support that post-conditioning activation of NMDA receptors containing GluRε2 in the striatum were crucial for the auditory fear memory formation with a lower-intensity footshock. This finding is consistent with the observation that GluRε2 predominates GluRε1/GluN2A in NMDA receptors of the striatum [20].

### Intra-striatum infusion of anisomycin impaired the formation of auditory fear memory

**Figure 4:**
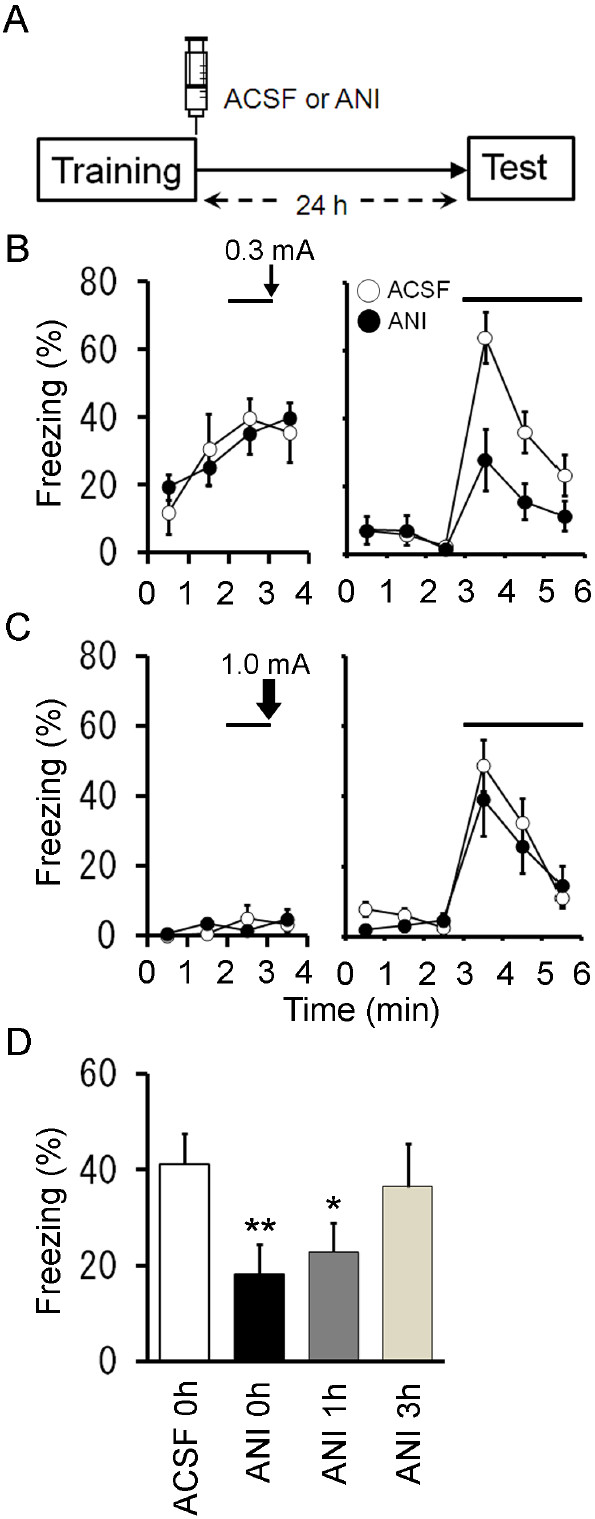
**Post-training infusion of anisomycin into the striatum impaired long-term fear memory. **(**A**) Experimental design. Mice were injected with 0.5 μl of ACSF or anisomycin into the striatum immediately after auditory fear conditioning, and tested 24 h later. (**B**) Freezing responses of ACSF (*n *= 8) and anisomycin (*n *= 7) groups on the conditioning at 0.3 mA (left) and test (right) days. Solid lines and a thin arrow represent tone and footshock, respectively. (**C**), Mice were injected with ACSF or ANI immediately after the conditioning or 1 h or 3 h after the conditioning (0.3 mA), and tested 24 h later. Bar graph shows the average freezing responses during tone presentation (3 min) of ACSF group with ACSF injections right after conditioning (ACSF 0 h; *n *= 8 as in Figure 5B), ANI groups with ANI injections immediately (ANI 0 h; *n *= 7 as in Figure 5B) or 1 h (ANI 1 h; *n *= 9) or 3 h (ANI 3 h; *n *= 6) after conditioning. The same data set as the ACSF group and ANI groups in (**B**) are also shown. *, *P *< 0.05; **, *P *< 0.01. (**D**), Mice were injected with ACSF or ANI into the striatum immediately after auditory fear conditioning with a high-intensity footshock (1.0 mA), and tested 24 h later. Freezing responses of ACSF (*n *= 7) and ANI (*n *= 7) groups on the conditioning (left) and test (right) days. Solid lines and a thick arrow represent tone and footshock (1.0 mA), respectively. ANI, anisomycin; BLA, basolateral amygdala; CeA, central amygdala; CP, caudate putamen.

We then asked whether protein synthesis in the striatum is involved in long-term fear memory. We infused anisomycin (62.5 μg in 0.5 μl) into the striatum immediately after conditioning (Figure 4A). The treatment with a protein synthesis inhibitor diminished freezing responses to the conditioned stimulus (CS) in the test 24 h following conditioning (Figure 4B; *F*_1,13_ = 9.3, *P* = 0.009, *n* = 8 (ACSF) or 7 (anisomycin), repeated measures ANOVA).

We then tested whether a time window also exists in the striatum, by delaying anisomycin infusions (62.5 μg) for 1 or 3 h after conditioning. Anisomycin infusion 1 h after conditioning produced a decrease in freezing in response to the CS in the test (Figure 4C; *F*_1,15_ = 5.15, *P* = 0.038, *n* = 9, compared to ACSF group in Figure 4B). In contrast, infusion 3 h after conditioning had no effect (Figure 4D; *F*_1,12_ = 0.22, *P* = 0.65, *n* = 6). It should be noted that the impairment produced by delaying anisomycin infusion is much smaller than that seen when anisomycin is given immediately after learning (Figure 4D). No effect of anisomycin treatment 3 h after training indicated that behavioral impairment by immediate post-training treatment cannot be explained as a side-effect of drug infusions. Thus, consolidation has a time window within 3 h which protein synthesis is required for the long-term fear memory. We further confirmed that the treatment of anisomycin did not affect the long-term fear memory trained with a high-intensity shock, as above (Figure 4C; *F*_1,12_ = 0.50, *P* = 0.49, *n* = 7 each).

**Figure 5:**
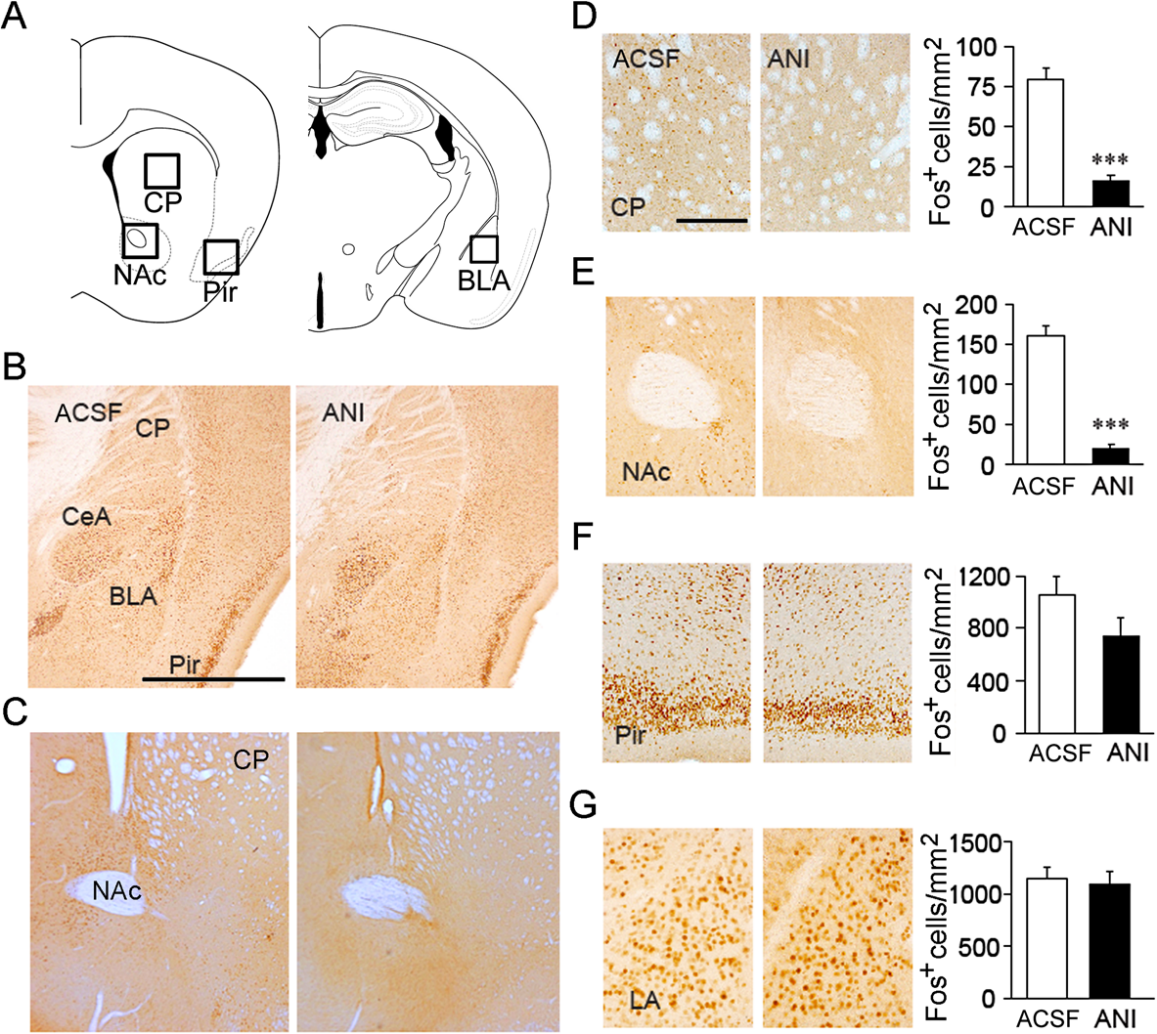
**Immunohistochemical analysis of Chloro-APB-induced Fos protein levels after anisomycin infusion into the striatum. **(**A**) Topographical organization of the CP, NAc, Pir and BLA. (**B, C**) Immunoreactivities to Fos in ACSF (left)- and anisomycin (right)-infused brains illustrate the extents of protein synthesis inhibition by anisomycin. High magnification images of Fos immunoreactivities in the CP (**D**), NAc (**E**), the Pir (**F**), and LA (**G**) in ACSF- and anisomycin-infused brains. Graphs are quantitative analysis of Fos-immunoreactive cell density. *n *= 8–10 slices from 3 mice each. ANI, anisomycin; BLA, basolateral amygdala; CeA, central amygdale; CP, caudate putamen; LA, lateral amygdala; NAc, nucleus accumbens; Pir, piriform cortex. Scale bar: 1.0 mm. (**B**); 0.2 mm (**D**). ***, *P *< 0.001.

We evaluated the effects of the anisomycin treatments on protein synthesis by Fos immunoreactivity in the CP, NAc, piriform cortex (Pir) and lateral amygdala (LA) (Figure 5A to C). Administration of anisomycin reduced Chloro-APB-induced Fos expression in the overlying CP and NAc significantly compared to control groups treated with ACSF (Figure 5D, E; *P* < 0.001, Mann-Whitney’s U test), indicating that anisomycin treatments successfully attenuated protein synthesis (Figure 4). This reduction in Fos expression was evident in a roughly circular region of 3 mm in diameter, around the tip of the injector. Anisomycin were diffused to neighboring structures, such as adjacent Pir (Figure 5F), but not to the amygdala (Figure 5G). In fact, anisomycin treatments hardly affected Fos expression in the LA. These results suggest that protein synthesis in the striatum within 3 h after conditioning is required for the long-term fear memory.

**Figure 6:**
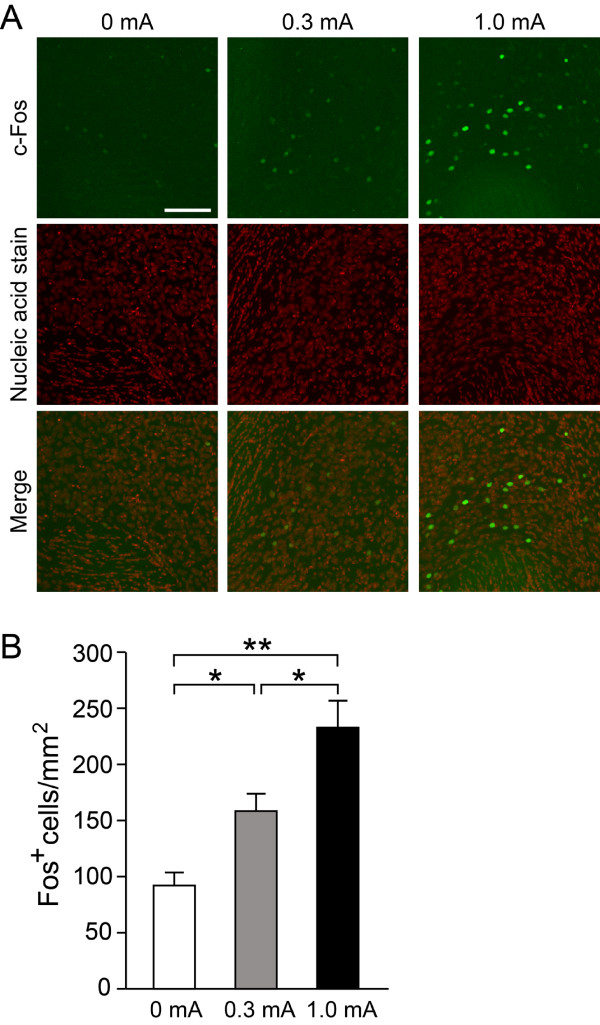
**Immunohistochemical analysis of c-Fos protein expression after auditory fear conditioning with different intensities of unconditioned stimuli in the striatum.** (**A**) Two hours after auditory fear conditioning with a footshock at 0 mA, 0.3 mA, or 1.0 mA, brains were sectioned and immunostained with anti-c-Fos antibody (upper panels, green). The nuclei were stained with SYTOX orange (middle panels, red). Representative images of coronal sections of NAc are shown as separate images (upper and middle panels) and merged images (lower panels). (**B**) Quantitative analysis of c-Fos-immunoreactive cell density in NAc. *n *= 18 slices from 3 mice each. Scale bar: 100 μm. * and **, *P *< 0.05 and *P *< 0.01.

We finally examined whether the US activates striatal neurons by measuring Fos immunoreactivities. Mice were subjected to auditory fear conditioning with or without footshocks. Fos-immunoreactive cell densities in the NAc increased according to the intensities of the US (Figure 6; *F*_2,51_ = 13.8, *P* < 0.001, one-way ANOVA). These results showed that footshocks activated striatal neurons in a dose-dependent manner.

## Discussion

We previously revealed that the ablation of striatal neurons induced in the adult brain impairs the long-term, but not short-term, memory for auditory fear conditioning with a lower-intensity footshock [15]. In the present study, we showed that the post-conditioning infusion of NMDA receptor antagonists in the striatum attenuated the conditioned fear trained with a lower-intensity footshock (0.3 mA). These results suggest that striatal NMDA receptors are involved in the memory consolidation rather than acquisition process. On the other hand, NMDA receptors in the amygdala are postulated to be involved in the acquisition process in the fear conditioning [6,21]. In the striatum, NMDA receptors are required for long-term potentiation and long-term depression, but play little role in synaptic transmission [22-25]. Striatal neurons receive massive glutamatergic afferents from the amygdala, cerebral cortex and hippocampus [26,27]. It is possible that the neuronal activities of other brain regions that store the short-term memory may induce NMDA receptor-dependent processes in the striatum during the post-conditioning consolidation period.

We also showed that the inhibition of protein synthesis in the striatum disrupted long-term fear memory when mice were trained with a lower-intensity footshock. There is considerable evidence that memory consolidation, the formation of a long-term memory, can be disrupted by a treatment of protein synthesis inhibitor given shortly after training, but that the same treatment given several hours or days later has no effect [7,28]. Our results suggest that the consolidation of auditory fear memory formed with a weak US requires NMDA receptors and protein synthesis in the striatum. However, NMDA receptor antagonists and protein synthesis inhibitor exerted little effects on the auditory fear conditioning with a high-intensity footshock (1.0 mA), in agreement with previous studies showing that the amygdala but not the striatum plays a central role in the auditory fear conditioning [1-4]. Thus, the involvement of the striatum in the auditory fear memory is selective for the conditioning with a lower-intensity footshock. The striatum plays a central role in integrating neural information from the cerebral cortex and thalamus to facilitate selection of actions that achieve reward-seeking outcomes and avoid aversive outcomes [29]. During auditory fear conditioning, striatal neurons are activated by the aversive stimulus in a dose-dependent manner (Figure 6). Despite the activation by footshocks, the striatum is not essential for auditory fear conditioning with standard US. It is likely that the amygdala system predominates over the striatal one when mice were conditioned with high-intensity footshocks. When the US becomes weaker, it will be less threatening and more difficult for animals to judge whether it is dangerous enough to be memorized. Our results suggest that the striatum is required for integrating weak threatening information into auditory fear memory formation.

## Conclusions

Post-conditioning activation of NMDA receptors and protein synthesis in the striatum are required for the memory consolidation of auditory fear conditioning at low-intensity shock. These results suggest that the striatum is crucial for the consolidation of auditory fear memory when US is weak.

## Materials and methods

### Animals

Male C57BL/6N mice (Clea Japan, Tokyo, Japan) of 8–12 weeks old (20–29 g) were used as subjects. Animals were individually housed under standard laboratory conditions with a 12-h day/night cycle; light was switched on at 8:00 and room temperature was maintained at 23 ± 1°C. Food and water were offered *ad libitum*. The experiments were performed during light phase. All animal procedures were approved by the Animal Care and the Use Committee of Graduate School of Medicine, the University of Tokyo (Approval #1721S062).

### Surgery

Surgery was conducted as described previously [17]. Mice were anesthetized with ketamine (80 mg/kg, i.p.; Sankyo Co., Tokyo, Japan) and xylazine (20 mg/kg i.p.; Bayer, Tokyo, Japan), and fixed to a stereotaxic apparatus (David Kopf, Tujunga, CA, USA). The animals were implanted two single guide cannulae (26 gauge; C315GS-5-SPC, Plastics One, Roanoke, VA, USA) into the NAc bilaterally at an angle of ±10° (stereotaxic coordinates: AP = +1.3 mm from bregma, ML = ±1.1 mm from midline, DV = -3.6 mm from bregma), according to an atlas of mouse brain [30]. The tip of internal cannula (33 gauge; C315DCS-5-SPC, Plastics One) for microinjection was inserted 1 mm below the tip of the guide cannulae (ML = ±1.0 mm, DV = -4.5 mm). The cannulae were fixed to the skull with dental cement. The animals were allowed to recover for at least 5 days before conditioning experiments.

### Drugs and infusions

Artificial cerebrospinal fluid (ACSF) was consisted of 150 mM NaCl, 3 mM KCl, 1.4 mM CaCl_2_, 0.8 mM MgCl_2_, 0.8 mM Na_2_HPO_4_, and 0.2 mM NaH_2_PO_4_ (pH 7.4). DL-2-Amino-5-phosphonovaleric acid (APV) (Sigma-Aldrich, MO, USA) was dissolved in ACSF at a concentration of 10 mM [1,17]. Ifenprodil (Sigma-Aldrich) was dissolved in distilled water and then diluted with ACSF to produce a final concentration of 2 μg/μl. Anisomycin was dissolved in equimolar HCl, diluted with ACSF and adjusted to pH 7 with NaOH to produce a final concentration of 125 μg/μl. During drug infusions, mice were restrained lightly in the disposable vinyl jacket (Braintree Scientific, Inc, MA, USA). Drug or ACSF was infused at a rate of 0.2 μl/min using a microinjection pump (CMA/100, CMA/Microdialysis, Solna, Sweden). The infusion cannulae were left in place for a further 1 min to diffuse the drug from needle tip, and the animal then returned to its home cage. Mice were injected the micro-ruby solution (Invitrogen, Carlsbad, CA, USA) and perfused 30 min later to examine dye diffusion.

### Auditory fear conditioning

Fear conditioning was tested using computer-controlled fear conditioning system (CL-M2; O’Hara, Tokyo, Japan), as described previously [15]. For paired conditioning paradigm, mice were placed in the conditioning chamber for 2 min and then a loud tone (65 dB, 10 kHz) was presented for 1 min through a speaker on the ceiling of the conditioning chest. At the end of the tone presentation, the mice were given a scrambled electrical footshock (0.3 or 1.0 mA, 1 s). One minute after footshock, the mice were returned to their home cages. For unpaired conditioning, mice were received the footshock immediately after they placed in the conditioning chamber and 2.5 min after the placement, the loud tone was presented for 1 min. Twenty-four hours after conditioning, mice were placed in a novel translucent acryl chamber with paper chips surrounded by a sound-attenuating black chest for 3 min and subsequently the tone was presented for 3 min. Bilateral drug or ASCF infusions into the striatum took place when animals were taken from a conditioning chamber immediately after the conditioning, except that anisomycin was also injected 1 or 3 h after conditioning. Freezing behavior was defined as the absence of any visible movement of the body and vibrissae except for movement necessitated by respiration. Freezing time was summated and the percentage of freezing was calculated per minute [15].

### Histochemistry

Each animal received ACSF in one side of the striatum and anisomycin in the contralateral one. Mice were then systemically administered with selective D1 agonist Chloro-APB (Sigma-Aldrich, 5 mg/kg, i.p.) and returned to their home cages for 2 h. The effect of the D1 receptor agonist treatment was estimated by the induction of the immediate early gene Fos expression. Mice were transcardially perfused with 4% paraformaldehyde in 0.1 M phosphate buffered saline (PBS). Brains were removed and post-fixed overnight in the same fixative. Brains were cut into 50 μm-thick free floating sections on a vibrating microtome (Leica Microsystems, Wetzlar, Germany). Sections were treated with 0.3% H_2_O_2_ in PBS for 20 min, washed in PBS, incubated with blocking solution (10% normal goat serum in PBS) for 1 h and further incubated with anti-Fos antibody (1:500, Merck KGaA, Darmstadt, Germany) overnight at room temperature. After 3 washes in PBS, tissue sections were incubated with the biotinylated secondary antibody (Nichirei, Tokyo, Japan) for 2 h. After 3 washes in PBS, sections were incubated in streptavidin-conjugated peroxidase (Nichirei) for 1 h. Peroxidase was revealed by incubating sections with 0.5 mg/ml 3-3′-diaminobenzidine (Sigma-Aldrich) and 0.002% H_2_O_2_ in PBS for 2 min. Sections were then mounted on glass slides, dehydrated in ethanol solutions and xylene, and coverslipped. The numbers of Fos-positive cells were counted at the dorsolateral part of CP, the dorsomedial part of NAc core, the Pir (AP = 1.2 mm from bregma), and the LA (AP = -1.7 mm) in the coronal brain sections. Only unequivocally stained cells were counted using the ImageJ software by two observers blind to the origin of the sections.

Two hours after auditory fear conditioning with a footshock at 0 mA, 0.3 mA, or 1.0 mA, brain sections were prepared as described above. Sections were blocking with PBS containing 5% normal goat serum, 1% BSA, and 0.3% Triton-X for 1 h at room temperature and further incubated with rabbit anti-c-Fos antibody (1:10000, Oncogene Research Products, Cambridge, MA) overnight at 4°C. After washing, the sections were incubated with Alexa Fluor 488 goat anit-rabbit IgG (1:1000, Molecular Probes, Eugene, OR) and SYTOX Orange nucleic acid stain (1:15000, Molecular Probes) in PBS containing 0.3% Triton-X for 2 h at room temperature. Fluorescence images were taken with a confocal laser-scanning microscope (TCS SP5; Leica Microsystems, Wetzlar, Germany).

### Statistics

Data are Mean ± SEM. The statistics significance was evaluated using Mann-Whitney’s U test, repeated measures ANOVA or one-way ANOVA followed by Tukey’s *post hoc* test. The criterion for statistical significance was *P* < 0.05.

## Abbreviations

ACSF: Artificial cerebrospinal fluid; APV: DL-2-Amino-5-phosphonovaleric acid; BLA: Basolateral amygdla; CeA: Central amygdla; CP: Caudate putamen; CS: Conditioned stimulus; LA: Lateral amygdala; NAc: Nucleus accumbens; NMDA: *N*-methyl-D-aspartate; PBS: Phosphate buffered saline; Pir: Piriform cortex; US: Unconditioned stimulus.

## Competing interests

The authors declare no competing financial interests.

## Authors’ contributions

AK, TU and FF performed the experiments and analyzed the data. AK, TU, FF and MM designed the experiments and wrote the paper. All authors read and approved the final manuscript.
